# Nonfreezing Cold Injury (Trench Foot)

**DOI:** 10.3390/ijerph181910482

**Published:** 2021-10-06

**Authors:** Ken Zafren

**Affiliations:** 1 Department of Emergency Medicine, Alaska Native Medical Center, 4315 Diplomacy Drive, Anchorage, AK 99508, USA; zafren@stanford.edu; 2 Department of Emergency Medicine, Stanford University Medical Center, 900 Welch Road, Suite 340, Palo Alto, CA 94304, USA; 3 International Commission for Mountain Emergency Medicine (ICAR MedCom), 8058 Zürich, Switzerland

**Keywords:** nonfreezing cold injury, trench foot, immersion foot, pressure necrosis, wilderness medicine, thermal injury, immersion injury, peripheral neuropathy, complex regional pain syndrome, hyperhidrosis

## Abstract

Nonfreezing cold injury (NFCI) is a modern term for trench foot or immersion foot. Moisture is required to produce a NFCI. NFCI seldom, if ever, results in loss of tissue unless there is also pressure necrosis or infection. Much of the published material regarding management of NFCIs has been erroneously borrowed from the literature on warm water immersion injuries. NFCI is a clinical diagnosis. Most patients with NFCI have a history of losing feeling for at least 30 min and having pain or abnormal sensation on rewarming. Limbs with NFCI usually pass through four ‘stages.’ cold exposure, post-exposure (prehyperaemic), hyperaemic, and posthyperaemic. Limbs with NFCI should be cooled gradually and kept cool. Amitriptyline is likely the most effective medication for pain relief. If prolonged exposure to wet, cold conditions cannot be avoided, the most effective measures to prevent NFCI are to stay active, wear adequate clothing, stay well-nourished, and change into dry socks at least daily.

## 1. Introduction

Nonfreezing cold injury (NFCI) is a modern term for trench foot or immersion foot. Other conditions also caused by cold without freezing include chilblains (pernio), cold urticaria, cryoprecipitation, and Raynaud’s phenomenon. To avoid confusion, these conditions should not be referred to as NFCIs. Moisture is required to produce a NFCI. NFCIs most commonly occur in military operations and training, but can also affect civilians, especially those without homes. NFCI seldom, if ever, results in loss of tissue unless there is also pressure necrosis or infection. Infection can be associated with pressure necrosis. Much of the published material regarding management of NFCIs has been erroneously borrowed from the literature on warm water immersion injuries. Warm water immersion injuries are very different than NFCIs, requiring different treatment.

## 2. Methods

All published journal articles, book chapters, and guidelines relevant to nonfreezing cold injuries were eligible for inclusion as references. I performed a MEDLINE search using the terms: nonfreezing cold injury, trench foot, immersion foot, and immersion injury. I also manually searched the reference lists of eligible articles for additional articles.

## 3. Definitions

NFCI is synonymous with trench foot or immersion foot. Trench foot usually refers to a NFCI that occurs on land, even if the feet are immersed. Immersion foot is generally the result of a shipwreck in which the feet are immersed in water in a lifeboat or life raft for hours to days. NFCI is affects the nerves, microvasculature, and soft tissue of the distal limbs, most often the feet. NFCI can also affect the hands and other areas, such as the knees and buttocks of shipwrecked sailors in lifeboats or life rafts. A diver exposed to water at 6 °C sustained an NFCI to one hand [1].

Other names for NFCI, include ‘sea boot foot’ and ‘bridge foot’. These two terms describe NFCI in sailors who wore rubber sea boots while moving little for at least four hours [2].

## 4. Other Cold-Induced Conditions

### 4.1. Frostbite

Frostbite is a local cold injury caused by freezing of tissue. Exposure to temperatures at least several degrees below freezing is required to produce frostbite. Because moisture is required to cause NFCI, the conditions producing frostbite and NFCI are mutually exclusive. In changing conditions frostbite and NFCI can occur together or frostbite can affect tissue that has already sustained NFCI.

### 4.2. Chilblains (Pernio)

Chilblains (pernio) are localised inflammatory lesions. They can be caused by cold or can be a complication of inflammatory disease without exposure to cold, especially systemic lupus erythematosis or COVID-19. When chilblains are caused by inflammatory disease, the term ‘pernio’ is usually used. Chilblains can be caused by cold conditions above freezing, especially by prolonged or recurrent exposure to damp or dry cold. Lesions are often red or purple nodules or areas of edema. Lesions are most commonly painful and are sometimes also pruritic.

### 4.3. Cold Urticaria

Cold urticaria, sometimes called cold contact urticaria, is an allergic reaction to contact with cold that produces urticaria (hives) or angioedema.

### 4.4. Cryoprecipitation

In some individuals, blood proteins may precipitate out of solution at temperatures colder than 37 °C. In cryoglobulinaemia, blood proteins precipitate from serum and plasma. Cryoglobulinaemia does not cause symptoms but can result in hyperviscosity or thrombosis. Cryofibrinogenaemia is caused by precipitation of proteins from plasma. Cryofibrinogenaemia is generally asymptomatic but can cause thrombosis.

### 4.5. Raynaud’s Phenomenon

Raynaud’s phenomenon is extreme, but reversible, vasoconstriction of the distal areas of digits in response to cold or to emotional distress. Distal parts of digits transiently develop well-demarcated pallor, cyanosis, or both.

## 5. History

NFCI was first described in Napoleon’s winter campaign in Russia in 1812 [3]. The term ‘trench foot’ was introduced in the First World War [4]. The term ‘immersion foot’ was first used in the Second World War to describe cold water immersion injuries in shipwrecked sailor in lifeboats [5]. US armed forces sustained 11,000 cases of trench foot in November 1944 during the Second World War in the European theatre [6]. In 1982, during the Falklands conflict, one UK brigade had a 76% incidence of NFCI [6].

## 6. Epidemiology

Most cases of NFCI have been described in victims with cold, wet extremities for at least one to three days [4,7], but NFCI can develop after 14 to 22 hours of exposure to sea water at 0 to 8 °C [2]. NFCI typically occurs in wet, cold conditions in victims who are unable to remove their shoes or boots whilst they are relatively immobile. Victims are also usually fatigued and calorie-depleted.

Statements that NFCI can occur in warm or dry conditions [6] and that exposure to temperatures as warm as 20 °C or exposures shorter than one hour can result in NFCI [8] are not supported by evidence.

NFCI is most likely to occur in military combat operations and training. Civilians can also be affected, especially those in occupations that can lead to long periods with wet feet, such as fish processers and harbour workers. Exposure to cold wet conditions without replacing wet socks and boots can cause NFCI in recreational activities, such as hiking and mountaineering, and in association with homelessness or alcoholism. Elderly persons are at greater risk than the general population. Reports that certain ethnic groups have increased susceptibility to NFCI have not been substantiated [2,9].

Most NFCIs currently being seen in clinics seem to be less severe than those described in the past. The aetiology of this change is not known. Advances in clothing and better prevention of NFCIs may be significant factors. Another change may be better conditions during wartime. Trench warfare is mostly of historical interest. There have been few conflicts fought in cold, wet conditions since the Second World War and the Falklands campaign. Because there is no standard for diagnosing NFCI, many patients diagnosed with NFCIs may actually have other conditions with milder symptoms.

## 7. Risk Factors

As with many environmental conditions, people vary greatly in vulnerability to NFCI. Immobility and inability to dry socks and boots are likely the most important risk factors. Associated risk factors include insufficient or wet clothing, footwear and clothes that are too tight, fatigue, stress, and deficient intake of calories or fluids. Conditions associated with vasculopathies, such as peripheral vascular disease, diabetes, and Raynaud’s phenomenon may lead to increased risk of NFCI. Older age, ethnicity, and smoking may also cause increased risk. Conditions that impair judgment, such as psychosis or intoxication, especially with alcohol, can also contribute to the development of NFCI.

A military study from the UK found that of 100 soldiers seen at a military clinic specialising in NFCI, only 76 had a diagnosis of NFCI. The remaining 24 soldiers had other diagnoses, especially neuropraxia and Raynaud’s phenomenon [10]. NFCI was most common during winter training exercises and in younger, unseasoned soldiers. Other factors increasing the risk of NFCI were immersion in water, wet boots and clothing, activities not involving movement, and ‘feeling generally cold.’ Afro-Caribbean troops seemed to be at greater risk than Caucasian troops. Smoking was not found to be a risk factor.

## 8. Physiology

Skin is essential for thermoregulation. The skin uses blood flow to regulate heat loss from the body. Skin blood flow is regulated by skin temperature, modulated by the temperature of blood flowing to the hypothalamus of the brain. Vasoconstriction can limit skin blood flow to about 10% of baseline. Because skin has minimal metabolic demands, vasoconstriction does not cause ischaemia. Skin blood flow in dependent extremities can also be decreased by immobility.

Vasoconstriction is maximal in the hands and feet at a skin temperature of about 15 °C. When skin cools below 15 °C, cold-induced vasodilation (CIVD) causes periodic increases in blood flow. CIVD, also known as the ‘hunting response,’ takes place in cycles lasting five to ten minutes. The cycles are more rapid and involve larger changes in blood flow in people who have regular, long-duration cold exposure. Inuits, Lapps, and fishermen in Nordic countries generally have strong, rapid CIVD cycles [11]. Theoretically, a vigorous CIVD response may decrease the likelihood of NFCI. In a study of 26 Dutch Marines, the authors concluded that weak CIVD response was associated with high risk of cold injury [12], but this conclusion was based on 11 subjects who subsequently were frostbitten. None of the subjects developed NFCI in follow-up.

## 9. Pathophysiology

The pathophysiology of NFCI is not well established. The predominant manifestations are dysfunction of circulatory control and injury to the microcirculation Animal studies have shown thrombosis with endothelial injury to the microcirculation. Reperfusion injury is probably a contributory mechanism. Animal studies also suggest the possibility that transient or permanent nerve injury with microvascular injury affects perfusion to nerves. Victims of severe hypothermia have suffered reversible peripheral nerve injury [13,14].

The idea that NFCI is caused by sustained vasoconstriction with ischaemia [6,8] is probably too simplistic [15]. In animal models, tissue injury is more severe with colder tissue temperatures and longer exposures. Intermittent short exposures cause greater injury than one longer exposure [6,8]. None of these effects has been quantified. NFCI has not been shown to occur with exposure to dry cold. Moisture seems to be a necessary factor. Immobility and inadequate intake of food and fluids also play a role in many, if not most, cases.

Tissue loss probably does not occur in uncomplicated NFCIs. Tissue loss associated with NFCI is most likely a sequela of pressure necrosis, or occasionally of compartment syndrome, when boots constrict the feet after swelling. Cold wet conditions with foot swelling can also lead to mechanical tissue damage, especially in victims who are forced to walk.

## 10. Diagnosis 

NFCI is a clinical diagnosis. Most patients with NFCI have a history of losing feeling for at least 30 min and having pain or abnormal sensation on rewarming [10]. To diagnose NFCI there must be a history of exposure to wet cold for at least several hours in temperatures near freezing or an exposure for days with higher temperatures, as high as about 15 °C. NFCI may be associated with frostbite if there was also exposure to temperatures well below freezing. Both frostbite and NFCI may present with red, oedematous hands or feet. Unlike frostbite, which is sharply demarcated between frostbitten areas distally and unfrozen areas proximally, sharp demarcation is not seen with NFCI.

### 10.1. Stages of NFCI

Limbs with NFCI usually pass through a sequence of four stages [2,8]. The lengths of the stages vary widely amongst victims. Some stages may be very short and easy to miss. The transition time from one stage to another may be short or long.

**Figure 1:**
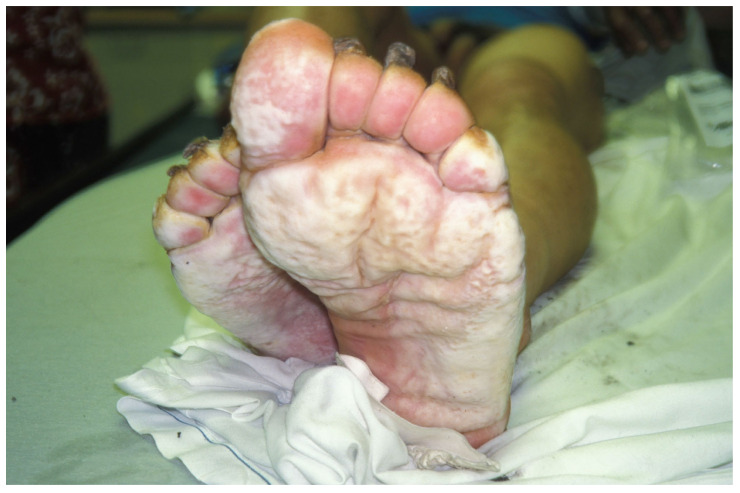
Nonfreezing cold injury (trench foot). Photo courtesy of Ken Zafren.

The first stage—cold exposure—is characterised by complete loss of sensation. Victims describe numbness or having feet or hands feel like blocks of wood. Because sensation and proprioception are lost, it is not unusual for victims to have trouble walking. Limbs may be bright red initially, but soon become pale or white because of severe vasoconstriction (Figure 1). This stage is generally painless.

The second-prehyperaemic (or post-exposure)-stage—starts when the victim is rescued from cold exposure and placed in a warmer environment. This stage takes place during and following rewarming. The duration is extremely variable, from a few hours to several days. In light-skinned victims, the skin appears mottled and pale blue, indicating the return of circulation at a very low level. This color change can be difficult to see in victims with darkly pigmented skin. Pulses are weak in the early part of the second stage, but later become strong. Slow capillary refill persists, however. The limbs remain cold and insensate. There may be swelling.

The third-hyperaemic-stage usually starts suddenly then persists for days or for as long as 10 weeks in severe cases. The limb is bright red and swollen with strong pulses. Delayed capillary refill persists because of injury to the microcirculation. Numbness is replaced by extreme pain with hyperalgesia, although some distal areas may still be insensate. There is usually no tissue damage. Blisters may arise in injured areas that have suffered pressure injury or infection. Although tissue loss is rare, areas of blistering or discoloration may signify incipient necrosis.

The fourth-post-hyperaemic-stage may last for weeks to years or be permanent. The limb has a normal appearance except in rare cases where tissue has been lost. Limbs are cool and are usually exquisitely cold-sensitive. Commonly, limbs vasoconstrict when exposed to cold. Limbs may stay cold for hours, even after very brief cold exposure. Chronic pain in response to cold is common. Victims often complain of excessive sweating (hyperhidrosis). Victims may develop symptoms that resemble complex regional pain syndrome (CRPS). Tissue necrosis, although rare, can lead to amputation in extreme cases.

### 10.2. Differential Diagnosis of NFCI

Frostbite is caused by temperatures at least several degrees below freezing, especially by exposure to cold air. NFCI cannot occur without moisture, so NFCI does not occur at temperatures much below freezing. However, frostbite and NFCI can coexist in some cases. Both can make the skin appear pale initially, but frostbitten tissue that is still frozen has a waxy appearance and a firm or hard consistency, unlike NFCI, in which tissue does not freeze. 

Limbs with superficial frostbite can become red and swollen after thawing, but areas with deep frostbite become cyanotic and demarcate from unaffected tissue or tissue with superficial frostbite. Development of blisters within 24 hours is typical of frostbite. Blisters do not occur in NFCI unless tissue becomes necrotic from pressure injury or infection.

Pressure necrosis, including acute compartment syndrome, is caused by elevated tissue pressure that limits circulation to the affected tissue. Ischaemia is intensely painful, usually out of proportion to physical exam findings. Acute compartment syndrome can also cause ischaemia in parts of limbs that are distal to the local area of increased tissue pressure. Pressure necrosis can be associated with NFCI when swelling causes footwear and clothing to be constrictive. Pressure necrosis associated with NFCI is usually insidious, because the affected areas are insensate. Tissue necrosis most likely does not occur in uncomplicated NFCI. When pressure-induced necrosis is caused by immobility without cold exposure, necrosis usually is found over bony prominences. Pressure necrosis associated with NFCI generally affects soft tissue that is not associated with bony prominences.

Soft tissue infection can occur in association with NFCI from exposure to wet, cold conditions. In dry cold conditions, soft tissue infection could be mistaken for NFCI. Soft tissue infection may cause fever and systemic symptoms such as nausea. Low-grade fever can also be a feature of NFCI without infection [16]. NFCI often causes bilaterally symmetric injury, while infections are usually asymmetric. Advanced imaging can detect abscesses but is unlikely to determine whether inflammation of the soft tissues is caused by NFCI or by infection.

### 10.3. Investigations

The diagnosis of NFCI is made clinically. Imaging and laboratory testing are not helpful in most cases. In trauma or possible trauma, plain radiographs of hands or feet are usually indicated. Trauma to the foot may require computed tomography (CT) to find fractures that are occult on plain radiographs. If infection is a possibility, CT with contrast or magnetic resonance imaging (MRI) can be used to find an abscess, free air, or other stigmata of infection.

CT angiography, magnetic resonance angiography, and nuclear medicine scans are useful for staging of frostbite but are not useful for NFCI. Thermal imaging (infrared thermography) is not useful acutely but can be used to assess the severity of injury. Thermography was previously used by the UK armed forces, although supporting data were lacking [8].

## 11. Treatment

### 11.1. Prehospital Treatment

A victim should be moved to a warm environment as quickly as possible. A victim in the prehyperaemic stage should be carried rather than walk [2]. The victim should be wrapped in a vapour barrier with insulation, even over wet clothing. The wet clothing can be removed once the victim is in a warm environment. 

### 11.2. Emergency Department Treatment

If the victim is hypothermic, this should be treated before treating frostbite or NFCI. Frostbite that is still frozen should be treated by rewarming in water at 37–39 °C. If frostbite is not present, limbs with NFCI should not be rewarmed. Rubbing an affected limb, with or without snow can cause damage to skin and is never indicated. 

Fluid losses should be replaced with isotonic crystalloid (normal saline or lactated Ringer’s solution) by the intravenous or intraosseous route. Fluids should be warmed to 42 °C before infusion, to prevent further heat loss.

Limbs with NFCI should be rewarmed gradually, with rest, elevation, and gentle pat drying at room temperature [17]. Rapid rewarming can cause severe pain, increased oedema, and increased tissue ischaemia [2]. The victim should receive a tetanus booster according to usual guidelines. Prophylactic antibiotics are not needed for NFCI without trauma. Pain control may be necessary. Prophylaxis of venous thromboembolism may be indicated.

### 11.3. In-Hospital Treatment

Emergency department treatment should be continued. Limbs with NFCI should be elevated above heart level. Dressings should be loose to protect the circulation. The ideal treatment is to leave affected limbs open to air. 

Once the limb has warmed and has entered the hyperaemic (third) stage, is no longer insensate. Prophylactic treatment of pain before rewarming is not effective. Limbs should be cooled using a fan in a cool room at temperatures of 15 to 18 °C [2]. A small fan on a bedside table can be aimed at the feet. In the past, some authors recommended cooling the feet using ice bags insulated from the skin with sterile towels [16]. Although this treatment was reported to relieve swelling and pain, there is a risk of aggravating the injury or causing frostbite.

Pain relief with analgesics, including nonsteroidal anti-inflammatory drugs and opioids is generally not very effective. Vasodilators, including nifedipine, are also not effective [8]. Regional anaesthesia (lumbar sympathectomy) was sometimes used in the past for treatment of pain [6], but is now an obsolete procedure that is not recommended.

The UK armed forces use amitriptyline (50 to 100 mg orally at bedtime) to treat pain from NFCI, with higher doses for breakthrough pain [8]. Amitriptyline should be started at the onset of pain [18,19]. Gabapentin can be added or substituted when amitriptyline does not provide adequate pain relief. There have been no clinical trials.

Mild fever in the first 12 to 36 hours is common and usually transient [16]. Antibiotic treatment, to cover staphylococci, streptococci, and pseudomonas, is indicated if cellulitis occurs. Surgical consultation should be obtained if there are signs of tissue necrosis, such as the formation of proximal hemorrhagic blisters.

A high-protein diet may be helpful. Smoking should be forbidden.

### 11.4. Long-Term Treatment

Neuropathic pain and CRPS are common sequelae of NFCI. In one reported case, iloprost, a vasodilator that is an analog of prostaglandin I_2_, was used to treat a 41-year old military veteran who suffered from foot pain with decreased mobility 20 years after sustaining a NFCI [20]. A five-day infusion of iloprost led to a month of decreased pain with increased mobility. These benefits then waned for several weeks. Three months later, another infusion was given, causing increased pain that gradually came back to baseline in a few months.

After hospital discharge, UK soldiers with NFCI may be rehabilitated. They are allowed to work outdoors if they have only minor symptoms without numbness. Soldiers with peripheral neuropathy attend a clinic for further investigations, including measurement of intraepidermal nerve fibre density. Soldiers with chronic pain are referred to a pain management clinic. Soldiers who recover over time can be exposed to progressively colder environments as long as they remain asymptomatic with normal physical findings. Some soldiers can be returned to full duty if they respond normally to cold.

## 12. Complications

NFCI can be complicated acutely by tissue necrosis, or infection. On resuming walking, victims may have a ‘slapping, flat-footed, springless gait.’ This usually resolves in about one week [16].

During the third stage of NFCI (hyperaemic stage), victims develop severe pain with hyperalgesia to light touch (allodynia) without evidence of damage to tissue [8]. The pain may be worse at night. Analgesic medications, such as opioids and nonsteroidal anti-inflammatory drugs, are usually not effective. 

Peripheral neurovascular injury with abnormal sympathetic tone is the main cause of long-term complications [8]. More severe injuries usually produce more severe sequelae. Sequelae are similar to those of frostbite [21], but are often more debilitating. Sequelae are usually permanent unless the original injury was mild.

Limbs affected by NFCI often feel cold [10]. Persistent vasoconstriction is common, especially after cold exposure [3]. Cold exposure is usually painful [16], because of sensory or vaso-neuropathy [22,23]. In severe cases, exposure to cold can cause allodynia [24]. Walking may cause pain [16]. Persistent pain and allodynia may lead to the development of CRPS. 

Affected limbs may may have intermittent nail loss and skin ulcers without clear precipitating injuries. Severe arthropathy of major joints has also been described.

Excessive sweating (hyperhidrosis), in response to cold, heat, or emotional stimuli [8], is a common sequela. Hyperhidrosis may cause recurrent paronychial infections caused by fungi, resulting in thick, deformed fingernails and toenails. Pain, paraesthesias, Raynaud’s syndrome, and chronic fungal infections may develop after many years [8]. Chronic, incompletely controlled, pain, can cause psychiatric sequelae, such as depression, suicidal thoughts, and abuse of alcohol or other substances.

Some victims of NFCI can no longer work outdoors [6]. In severe cases, military personnel may be unable to redeploy, especially to cold environments. NFCI was career-ending for 25 of 42 soldiers who attended a pain clinic for sequelae of NFCI [22]. Other 17 soldiers required changes in duty to avoid exposure to cold. 

## 13. Prevention of NFCI

Specific measures to prevent NFCI have not been studied. Avoidance of wet cold is the best way of preventing NFCI. Some suggested methods have been extrapolated from warm water immersion injuries. Air drying of feet for >8 hours a day is effective in preventing warm water immersion foot [17,25]. A recommendation to dry feet for a day after every 2 days of immersion pertains to tropical immersion foot [26].

In the First World War, the Allied forces eliminated trench foot by increasing rations and providing dry socks in waterproof bags every night to troops in the trenches [7]. Other effective measures against NFCI included a ban on puttees, wraps around the calf and ankle above boots, to prevent constriction, encouraging soldiers to remain as active as possible, and the use of gum boots with foot powder instead of using oils. Use of grease or oils probably increases the risk of NFCI [7].

General measures to prevent NFCI include limiting exposure to cold, wet conditions and not exposing casualties to cold who have previously suffered NFCI. Clothing should be warm, even when wet. Synthetics are better than wool. Cotton should not be worn, as it is very cold when wet. Civilians, as well as military personnel, should be active to encourage circulation in the limbs. Feet should be elevated whenever possible. Stress can cause vasoconstriction. Education and training for cold can help to prevent or minimise stress. Rotating personnel in and out of cold environments is likely the most effective measure [3]. In one study, some soldiers were assigned in pairs to examine each other’s feet and boots on a frequent basis. Soldiers in pairs removed their boots and dried their socks and feet more often than soldiers who were not paired [23].

## 14. Conclusions

NFCI is caused by wet cold. Tissue loss only occurs if NFCI is complicated by pressure necrosis or infection. The mainstays of treatment are gradual rewarming and keeping affected limbs cool. The most important preventive measures are avoidance of prolonged exposure to wet cold conditions, maintenance of adequate insulation and nutrition, and regularly changing into dry socks.

## References

[1] Laden G.D., Purdy G., O’Rielly G. (2007). Cold injury to a diver’s hand after a 90-min dive in 6 degrees C water. Aviat. Space Environ. Med..

[2] Ungley C.C., Channell G.D., Richards R.L. (1946). The immersion foot syndrome. Brit. J. Surg..

[3] Francis T.J. (1984). Non freezing cold injury: A historical review. J. R. Nav. Med. Serv..

[4] Smith J.L., Ritchie J., Dawson J. (1915). On the pathology of trench-frostbite. Lancet.

[5] White J.C. (1943). Vascular and neurologic lesions in survivors of shipwreck. N. Engl. J. Med..

[6] Whayne T.F., DeBakey M.E. (1958). Cold Injury, Ground Type.

[7] Haller J.S. (1990). Trench foot—A study in military-medical responsiveness in the Great War, 1914–1918. West. J. Med..

[8] Thomas J.R., Oakley H.N., Pandolf K.B., Burr R.E. (2001). Nonfreezing cold injury. Medical Aspects of Harsh Environments.

[9] Webster D.R., Bigelow W.G. (1952). Injuries Due to Cold, Frostbite, Immersion Foot and Hypothermia. Can. Med. Assoc. J..

[10] Kuht J.A., Woods D., Hollis S. (2019). Case series of non-freezing cold injury: Epidemiology and risk factors. J. R. Army Med. Corps.

[11] Greenfield A.D., Shepherd J.T., Whelan R.F. (1951). Cold vasoconstriction and vasodilatation. Ir. J. Med. Sci..

[12] Daanen H.A., van der Struijs N.R. (2005). Resistance Index of Frostbite as a predictor of cold injury in arctic operations. Aviat. Space Environ. Med..

[13] Collier T., Patel A., Rinaldi R. (2012). Hypothermia-induced peripheral polyneuropathy after an episode of drowning. PM&R.

[14] Loseth S., Bagenholm A., Torbergsen T., Stalberg E. (2013). Peripheral neuropathy caused by severe hypothermia. Clin. Neurophysiol..

[15] Montgomery H., Horwitz O., Peirce G., Sayen A. (1954). Experimental immersion foot. I. The effects of prolonged exposure to water at 3 degrees C. on the oxygen tension and temperature of the rabbit leg. J. Clin. Investig..

[16] Webster D.R., Woolhouse F.M., Johnston J.L. (1942). Immersion foot. J. Bone Jt. Surg. Am..

[17] Wrenn K. (1991). Immersion foot. A problem of the homeless in the 1990s. Arch. Intern. Med..

[18] Aldington D.J., McQuay H.J., Moore R.A. (2011). End-to-end military pain management. Philos. Trans. R. Soc. Lond. B Biol. Sci..

[19] McGreevy K., Bottros M.M., Raja S.N. (2011). Preventing Chronic Pain following Acute Pain: Risk Factors, Preventive Strategies, and their Efficacy. Eur. J. Pain Suppl..

[20] Ionescu A.M., Hutchinson S., Ahmad M., Imray C. (2017). Potential new treatment for non-freezing cold injury: Is Iloprost the way forward?. J. R. Army Med. Corps.

[21] Mills W., Pandolf K., Burr R. (2001). Clinical Aspects of Freezing Cold Injuries. Medical Aspects of Harsh Environments.

[22] Vale T.A., Symmonds M., Polydefkis M., Byrnes K., Rice A.S.C., Themistocleous A.C., Bennett D.L.H. (2017). Chronic non-freezing cold injury results in neuropathic pain due to a sensory neuropathy. Brain.

[23] Anand P., Privitera R., Yiangou Y., Donatien P., Birch R., Misra P. (2017). Trench Foot or Non-Freezing Cold Injury As a Painful Vaso-Neuropathy: Clinical and Skin Biopsy Assessments. Front. Neurol..

[24] Jorum E., Opstad P.K. (2019). A 4-year follow-up of non-freezing cold injury with cold allodynia and neuropathy in 26 naval soldiers. Scand. J. Pain.

[25] Buckels L.J., Gill K.A., Anderson G.T. (1967). Warm water immersion foot. Res. Rep. US Nav. Med. Field. Res. Lab..

[26] Taplin D., Zaias N., Blank H. (1967). The role of temperature in tropical immersion foot syndrome. JAMA.

